# Identification of HnRNPC as a novel Tau exon 10 splicing factor using RNA antisense purification mass spectrometry

**DOI:** 10.1080/15476286.2021.2015175

**Published:** 2021-12-29

**Authors:** Sansi Xing, Jane Wang, Ruilin Wu, Marco M. Hefti, John F. Crary, Yu Lu

**Affiliations:** a Department of Biochemistry and Biomedical Sciences, McMaster University, Hamilton, ON, Canada; b Department of Medicine, McMaster University, Hamilton, ON, Canada; c Department of Pathology, University of Iowa, Iowa City, IA, USA; d Department of Pathology and Department of Neuroscience, Neuropathology Brain Bank & Research Core, Icahn School of Medicine at Mount Sinai, New York, NY, USA

**Keywords:** RNA antisense purification, mass spectrometry, Tau, alternative splicing, RNA-binding protein, hnRNPC, neurodegeneration, progressive supranuclear palsy

## Abstract

Alternative splicing in Tau exon 10 generates 3 R- and 4 R-Tau proteoforms, which have equal abundance in healthy adult human brain. Aberrant alternative splicing in Tau exon 10 leads to distortion of the balanced 3 R- and 4 R-Tau expression levels, which is a causal factor to trigger toxic Tau aggregation, neuron dysfunction and patient death in a group of neurodegenerative diseases known as tauopathies. Hence, identification of regulators upstream of the Tau exon 10 splicing events are crucial to understanding pathogenic mechanisms driving tauopathies. In this study, we used RNA Antisense Purification with Mass Spectrometry (RAP-MS) analysis to identify RNA-binding proteins (RBPs) that interact with the Tau pre-mRNA near exon 10. Among the newly identified RBP candidates, we show that knockdown of hnRNPC induces Tau exon 10 skipping whereas overexpression of hnRNPC promotes Tau exon 10 inclusion. In addition, we show that hnRNPC interacts with the poly-uridine (U-tract) sequences in introns 9 and 10 of Tau pre-mRNA. Mutation of these U-tract motifs abolished binding of hnRNPC with Tau pre-mRNA fragment and blocked its impact on Tau exon 10 inclusion. These findings indicate that hnRNPC binds and utilizes these U-tract motifs located in introns 9 and 10 of Tau pre-mRNA to promote Tau exon 10 inclusion. Intriguingly, high hnRNPC expression level is associated with progressive supranuclear palsy (PSP), a sporadic tauopathy with pathological accumulation of Tau species that contain exon 10, which suggests a putative therapeutic role of hnRNPC for PSP treatment.

**Figure:**
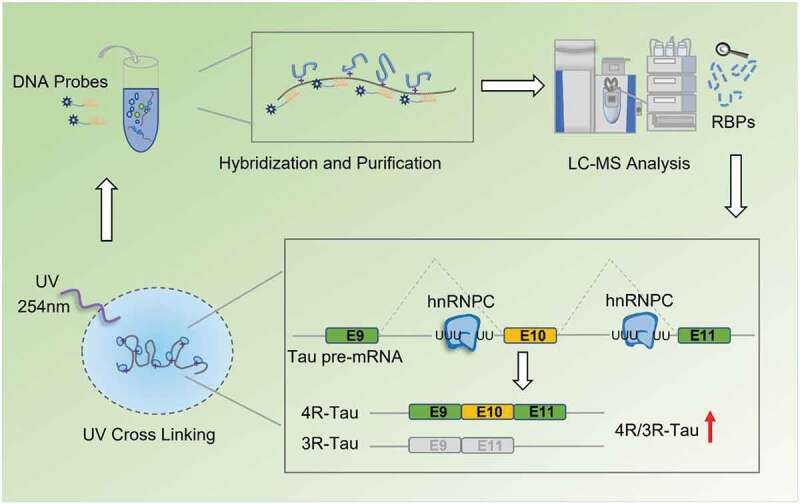

## Introduction

Tauopathies are a group of heterogeneous brain disorders with varying prevalence and symptoms that share the common feature of accumulation of toxic forms of the Tau protein in cytoplasmic aggregates [1]. Human Tau is encoded by the microtubule-associated protein tau (*MAPT*) gene located on chromosome 17. As a microtubule binding protein predominantly expressed in the nervous system, Tau plays a vital role in axonal transport and neuronal function [2]. Tau can form abnormal pathological aggregates in both neurons and glial cells in both sporadic and familial tauopathies [3–7]. The molecular drivers of the accumulation of abnormal forms of Tau are complex, with multiple factors contributing to the pathological process, including abnormalities in alternative splicing [8–13].

**Figure 1.**
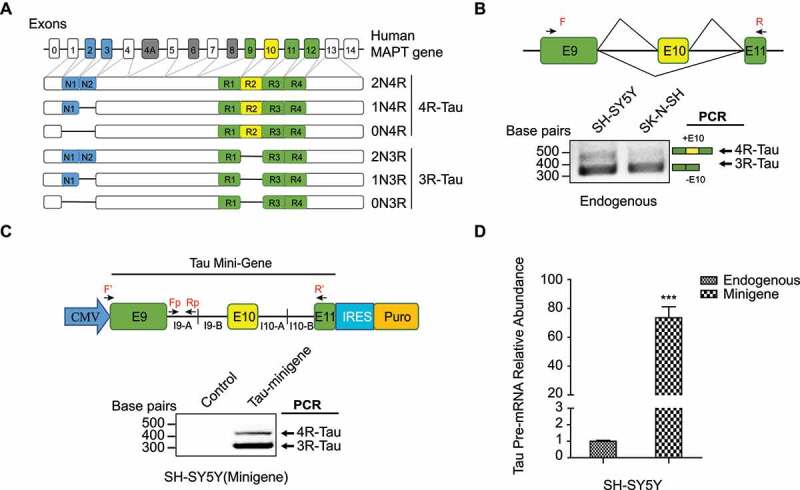
Expression of Tau isoforms. (a) human MAPT gene contains 16 exons. In the human central nervous system (CNS), there are six Tau isoforms, which are generated by alternative splicing in exons 2,3 and 10. Alternative splicing of exon10 gives rise to Tau with either 3 or 4 microtubule-binding domain repeats, termed 3 R- and 4 R-Tau respectively. Alternative splicing of exons 2 and 3 towards the N-terminus gives rise to Tau isoforms differ in the number of N-terminal inserts. (b) Endogenous 3 R- and 4 R-Tau isoforms in the SH-SY5Y and SK-N-SH neuroblastoma cell lines measured by RT-PCR, using primers F and R as shown in the top diagram. (c) Top: Construct of Tau minigene expressed in the neuroblastoma cell lines. I9-A: +1 to +990nt in intron 9; I9-B: +12,645 to +13645nt in intron 9; I10-A: +1 to +1000nt in intron10; I10-B: +2840 to +3840nt in intron 10. Bottom: 3 R- and 4 R-Tau isoforms expressed from the minigene measured using RT-PCR with the primers F’ and R’. (d) The Tau minigene pre-mRNA abundance through RAP process was measured by qRT-PCR using the primers Fp and Rp as shown in (c). Data shown represent the means ± SEM (n = 3, ***P < 0.001, two-tailed Student’s t test).

At the mRNA level, Tau exon 10 alternative splicing gives rise to Tau isoforms with either 3 or 4 microtubule-binding domain repeats, termed 3 R- and 4 R-Tau, respectively [14] (Figure 1(a)). Alternative splicing of exons 2 and 3, which encode amino acids towards the N-terminus of the resulting protein forms, gives rise to additional Tau isoforms mostly found in the central nervous system (CNS), namely 0N, 1 N, 2 N [14]. During brain development, a single isoform that lacks both alternatively spliced N-terminal exons and exon 10 (0 N3R) is the predominant species in the foetal human brain [15]. In the adult human brain, however, a combination of six major Tau isoforms is present (Figure 1(a)), where the ratio between the 3 R- and 4 R-Tau isoforms is relatively equal [16]. Aberrant alternative splicing of Tau exon 10 leads to an imbalance of Tau splicing isoforms and is strongly linked to several inherited tauopathies such as the frontotemporal dementia with parkinsonism linked to chromosome 17 (FTDP-17) [17]. FTDP-17 is associated with at least 39 *cis*-element mutations around the *MAPT* exon 10 region that leads to 3 R-Tau or 4 R-Tau enrichment in Tau aggregates [18,19]. Besides FTDP-17, in many other tauopathies, such as progressive supranuclear palsy (PSP) and Pick’ disease (PiD), post-mortem brain autopsies reveal that Tau aggregates are also preferentially enriched with 4 R-Tau and 3 R-Tau isoforms, respectively [20]. These tauopathies are therefore also termed as 4 R- and 3 R-tauopathies, accordingly. Importantly, most PSP cases are sporadic in nature, with little connection to genetic causes including mutations in the *MAPT* locus [21]. These findings suggest that *trans*-acting factors may play important roles in aberrant Tau exon 10 splicing in sporadic PSP and other 4 R- and 3 R-tauopathies. While many RNA binding proteins (RBPs) were discovered to be linked to Tau exon 10 splicing regulation [22–31], to date it remains uncertain how dysregulation of these known RBPs might contribute to the selective accumulation of 4 R-Tau or 3 R-Tau in PSP and other sporadic splicing-related tauopathies. Therefore, we hypothesize that there are additional unidentified splicing factors involved in mediating Tau exon 10 splicing.

Conventional methods to identify locus-specific splicing factors mainly rely on known splicing factor binding motifs [32] or *in vitro* RNA pull-down, combined with mass spectrometry (MS)-based proteomic analysis. However, for many splicing regulators, their specific RNA binding motifs are not known. Although *in vitro* RNA pull-down approaches combined with MS-based proteomics have been widely used to discover upstream splicing factors, these methods very often fail to detect the RNA-protein interactions that occur in live cells. *In vitro* RNA pull-down requires prior cell lysis and re-forming of RNA-protein complexes in buffer solution, which makes it difficult to preserve the native RNA-protein interactions. Furthermore, in these published *in vitro* RNA pull-down proteomic methods, both direct and indirect binding proteins are detected simultaneously, along with high background proteins, making the subsequent data interpretation and validation challenging [33]. The lack of effective and specific approach to comprehensively characterize protein interactors to a specific RNA splicing substrate in living cells remains a major obstacle in the RNA splicing field.

Recently, two oligo-based methods, Comprehensive identification of RBPs by MS (ChIRP-MS) [34], and RNA antisense purification by MS (RAP-MS) [35], were developed to identify RNA binding proteins that regulate long non-coding RNAs. These methods both avoid post-lysis formation of RNA-protein complexes and can therefore be used to detect *in vivo* RNA-protein interactions. Compared to the ChIRP-MS method, the RAP-MS method deploys longer probes (≥ 90nt) to tile the entire sequence of RNA to ensure robust capture. Importantly, the use of UV irradiation covalently links proteins to RNAs, making it possible for the subsequent probe-transcript hybridization step to occur at high temperature (> 67°C) to disrupt the RNA secondary structure for higher capture efficiency. In addition, this stringent condition with high temperature and high detergent/salt allows protein purification under denaturing conditions to remove undesired non-specific binding.

Given the promise to use RAP-MS to isolate specific RNA and its direct binding proteins in such a robust and sensitive way, in this study, we adapted and applied the RAP-MS method to detect RBPs that contribute to the Tau exon 10 alternative splicing event. Towards this end, we designed probes targeting the Tau pre-mRNA around its exon 10 region and performed RAP-MS proteomic analysis in the neuroblastoma cell line SH-SY5Y. Among the identified 15 novel RBP candidates, using loss of function assays, we identified 9 novel RBPs that function to mediate alternative splicing of Tau exon 10. Specifically, we fully validated the functional roles of hnRNPC in Tau exon 10 splicing regulation and characterized its functional binding sites in the Tau pre-mRNA sequence.

## Material and methods

### Plasmids and antibodies

pLKO.1 puro (#8453), pCDH-EF1(#72266), pCDNA3.1-FLAG-HA (#52535) were obtained from Addgene. Mouse monoclonal anti-hnRNPC (# ab128049, 1:1000 dilution) was from Abcam. Rabbit polyclonal anti-Tubulin (#2148S, 1:1000 dilution) was from Cell Signalling Technology.

### Cell culture

HEK 293 FT and HeLa were cultured in Dulbecco’s modified Eagle’s medium (DMEM) supplemented with 10% foetal bovine serum (FBS). The human neuroblastoma cell lines SH-SY5Y and SK-N-SH were cultured in Ham’s F12: Minimum Essential Medium Eagle (1:1), supplemented with 2 mM GlutaMAX, 1% Non-Essential Amino Acids (NEAA) and 10% FBS.

### Construction of Tau minigene plasmid

Tau minigene (4431 bp) was obtained by PCR amplification of three fragments from the genomic DNA of HEK 293 FT cells between MAPT exons 9–11 (Figure 1(c)), which were then seamlessly cloned together to the pCDNA3.1-FLAG-HA vector (Addgene, USA) in one step by using GeneArt® Seamless Cloning and Assembly Enzyme Mix (Thermo Scientific, USA).

### RT-PCR and quantitative real-time PCR

Total RNA from cells was extracted using the RNeasy®mini kit (Qiagen, USA) according to the manufacturer’s protocol and were used for both semiquantitative and quantitative PCR. Endogenous 3 R- and 4 R-Tau mRNA expression were detected by RT-PCR with the following primers: forward5ʹ- GCGAATTCGGTGAACCTCCAAAATCAGGGGATCG-3ʹ; reverse5ʹ-CCCTGGTTTATGTTGCCTAATGAG-3ʹ. Primers forTauminigene:forward5ʹ-GGTGGAGGTGGAGGTTCTAGAAGTG-3ʹ; reverse5ʹ- ACCTGAGCAAGG TGACCTCCA-3ʹ. RT-PCR products were detected on a 2% agarose gel: endogenous 3 R- and 4 R-Tau RT-PCR products were 359 and 452 bp, respectively. Tau minigene 3 R- and 4 R-Tau RT-PCR products were 334 and 427 bp, respectively. Gel bands were quantified with ImageJ and Significance was tested using two-tailed Student’s t test and p < 0.05 was considered significant. For quantitative real-time PCR (qRT-PCR), the RNA concentration was determined using a NanoDrop (Thermo Scientific, USA) and 2 μg RNA was reverse transcribed to cDNA using SuperScriptTM III First-Strand cDNA Synthesis. qRT-PCR was conducted with SsoAdvancedTM Universal SYBR Green Supermix (BIO-RAD, Mississauga, ON)). Primers used in qRT-PCR are shown in Table S1.

### Western blotting

Cells were collected and then lysed using 8 M urea and 100 mM ammonium bicarbonate on ice. Lysate protein concentrations were measured by micro BCA^TM^ protein assay kit (Thermo Scientific, USA). After normalization, equal amounts of proteins diluted in 1x LDS loading buffer containing 0.1 M DTT were boiled for 10 minutes at 95°C prior to electrophoresis. After gel electrophoresis, proteins were then transferred onto a nitrocellulose membrane using iBlot 2 Dry Blotting System (Thermo Scientific, USA), blocked with 5% skim milk in 1x TBST for 1 hour at room temperature and then incubated overnight at 4°C with primary antibodies. Following membrane washing, secondary antibodies IRDye 800 goat-anti-rabbit (Li-COR Biosciences, USA) or IRDye 800 goat-anti-mouse (Li-COR Biosciences, USA) were added for 1 hour at room temperature and washed three times with 1x TBST. The blot was then imaged with the Odyssey Classic Imager (Li-COR Biosciences, USA).

### Generation of recombinant lentivirus, stable cell lines, cell transduction

PLKO.1 lentiviral vector that expresses the scramble shRNA (as the negative control) was purchased from Sigma. The sequence of shRNA oligos that target individual Tau splicing factor candidates was selected based on Sigma’s pre-validation assays with the highest target efficiencies for each target. Meanwhile the sequence specificity for each oligo was confirmed using Primer-BLAST [36]. All these checked shRNA oligos were cloned into pLKO.1 lentiviral vector under the U6 promoter. Recombinant vectors and packaging plasmids pSPAX2 and pMD2.G were transfected into HEK 293 FT cells to produce recombinant lentivirus. To test the splicing profile of Tau exon 10, SH-SY5Y neuroblastoma cells were infected with lentivirus either expressing scramble-shRNA or candidates-shRNA followed by puromycin selection (2 µg/ml) for 48 hrs. The sequence for shRNA oligos used in this study is shown in Table S1.

### RNA antisense purification (RAP)

Probe design and generation: 90-mer DNA oligonucleotides spanning the target RNA with reverse complementary sequences were synthesized by Integrated DNA Technologies (IDT). Each DNA oligonucleotide probe carries a 5ʹ biotin for streptavidin capturing. Probes that may hybridize to off-target sequences or have melting temperature (Tm) below 67°C were removed. RAP oligos targeting the Tau minigene and the GAPDH mature mRNA are listed in Table S2.

UV cross linking: SH-SY5Y cells stably expressing the Tau-minigene were cultured in 150 mm dishes. Cells were UV irradiated with 600 mJ/cm^2^ in ice cold PBS at 80% cell confluence (~10 million cells per dish). Cells were then scraped, washed twice with PBS, and pelleted at 800 g for 5 min at 4°C.

Total cell lysate preparation: About 240 million cells (12x 20 million) were lysed by completely resuspending frozen cell pellets in ice-cold detergent-based cell lysis buffer (10 mM Tris pH 7.5, 500 mM LiCl, 0.5% dodecyl maltoside (DDM, Sigma, Oakville, ON), 0.2% sodium dodecyl sulphate (SDS, Sigma, Oakville, ON), 0.1% sodium deoxycholate (Sigma, Oakville, ON), 1× Protease Inhibitor Cocktail (Thermo Scientific, USA) and Murine RNase Inhibitor (New England Biolabs, Whitby, ON). Samples were then incubated for 10 minutes on ice to allow lysis to proceed. During this incubation period, each cell sample was passed five times through a 26-gauge needle attached to a 1 mL syringe to disrupt the pellets and shear genomic DNA. Each sample was then sonicated at 20% pulse power for 3 times in intermittent pulses (1 second on, 2 seconds off). The samples were then treated for 10 minutes at 37°C with 2.5 mM MgCl2, 0.5 mM CaCl2, and 20 U of TURBO DNase (Thermo Scientific, USA) to digest DNA. Samples were returned to ice and the reaction was immediately terminated by the addition of 10 mM EDTA and 5 mM EGTA. Disulphide bonds were reduced by addition of 2.5 mM Tris-(2-carboxyethyl) phosphine (TCEP) and samples were then mixed with twice the lysate volume of 1.5× LiCl/Urea Buffer (the final1 × Buffer contains 10 mM Tris pH 7.5, 5 mM EDTA, 500 mM LiCl, 0.5% DDM, 0.2% SDS, 0.1% deoxycholate, 4 M urea, 2.5 mM TCEP). Lysates were incubated on ice for 10 minutes then cleared by centrifugation for 10 minutes at maximum speed. Supernatants were saved.

RNA Antisense Purification of crosslinked complexes: Lysate samples from 240 million cells (half lysate used for Tau minigene pre-mRNA pull-down, the other half lysate used for GAPDH mature transcript control pull-down) were pre-cleared with 500 μl Streptavidin coated Sepharose beads (GE Health Care, Mississauga, ON) at 37°C for 30 minutes. Biotinylated 90-mer DNA oligonucleotide probes specific for the RNA target of interest (12 μg Tau probes and 12 μg GAPDH probes) were heat-denatured at 85°C for 3 minutes and then snap-cooled on ice. Probes and pre-cleared lysate were mixed and incubated at 67°C using Thermomixer R (Eppendorf, Mississauga, ON)) with intermittent shaking (1100 rpm, with 30 seconds on and 30 seconds off) for 2 hours. Hybrids of biotinylated DNA probes and target RNA were then bound to streptavidin beads at 67°C for 30 minutes with intermittent shaking as above. Beads with captured hybrids were washed 5 times with LiCl/Urea Hybridization Buffer at 67°C for 5 minutes to remove non-specifically associated proteins.

Elution and analysis of RNA samples: For qPCR examination of RNA captures, beads with hybrids were centrifuged at 500 g for 2 minutes at 4°C and then the supernatant was discarded. Beads were then resuspended by pipetting in 20 μL NLS RNA Elution Buffer (20 mM Tris pH 8.0, 10 mM EDTA, 2% NLS, 2.5 mM TCEP). To release the target RNA, beads were heated for 2 minutes at 95°C. Then eluted target RNA molecules were incubated with 1 μg Proteinase K (Thermo Scientific, USA) for 1 hour at 55°C to remove all proteins. The remaining nucleic acids were then purified by ethanol precipitation onto SILANE beads (Thermo Scientific, USA). DNA probes were removed by digestion with TURBO DNase (Thermo Scientific, USA). Further, qRT-PCR was performed to quantify RNA enrichment.

Elution and analysis of protein samples: For mass spectrometry analysis of captured protein contents, on beads trypsin digestion was performed. In brief, after oligo capturing, beads were further washed six times with 50 mM ammonium bicarbonate (pH = 8.0). 1.2 μg trypsin/ 120 million cells were added to the beads and incubated overnight at 37°C. 0.8 μg trypsin/120 million cells were added the next day and incubated for another 2–4 hrs. Trypsin Supernatant was collected for mass spectrometry analysis.

### Liquid chromatography and tandem mass spectrometry (LC-MS/MS)

Peptide samples were resuspended in 0.1% Trifluoroacetic acid (TFA), loaded onto a home-made trap-column (5 cm x 200 µm inner diameter; POROS 10um 10R2 C18 resin) and a home-made analytical column (50 cm x 50 µm inner diameter; Reprosil-Pur 120 C18-AQ 5 µm resin), and eluted with a 120 min reversed-phase gradient at 70 nl/min on a Thermo Fisher Ultimate 3000 RSLCNano UPLC (Ultra-Performance Liquid Chromatography) system coupled to a Thermo QExactive HF quadrupole-Orbitrap mass spectrometer. A parent ion scan was performed using a resolving power of 120,000 and then up to the 20 most intense peaks were selected for MS/MS. All raw data have been deposited in the ProteomeXchange Consortium via Proteomics Identification (PRIDE) [37]. The accession number of the proteomics data reported in this paper is PXD012432.

Data were analysed using Thermo Proteome Discoverer 2.2. For protein identification, search was against the SwissProt human proteome database (42,173 protein isoform entries), while the search parameters specified a parent ion mass tolerance of 10 ppm, and an MS/MS fragment ion tolerance of 0.02 Da, with up to two missed cleavages allowed for trypsin.

**Figure 2.**
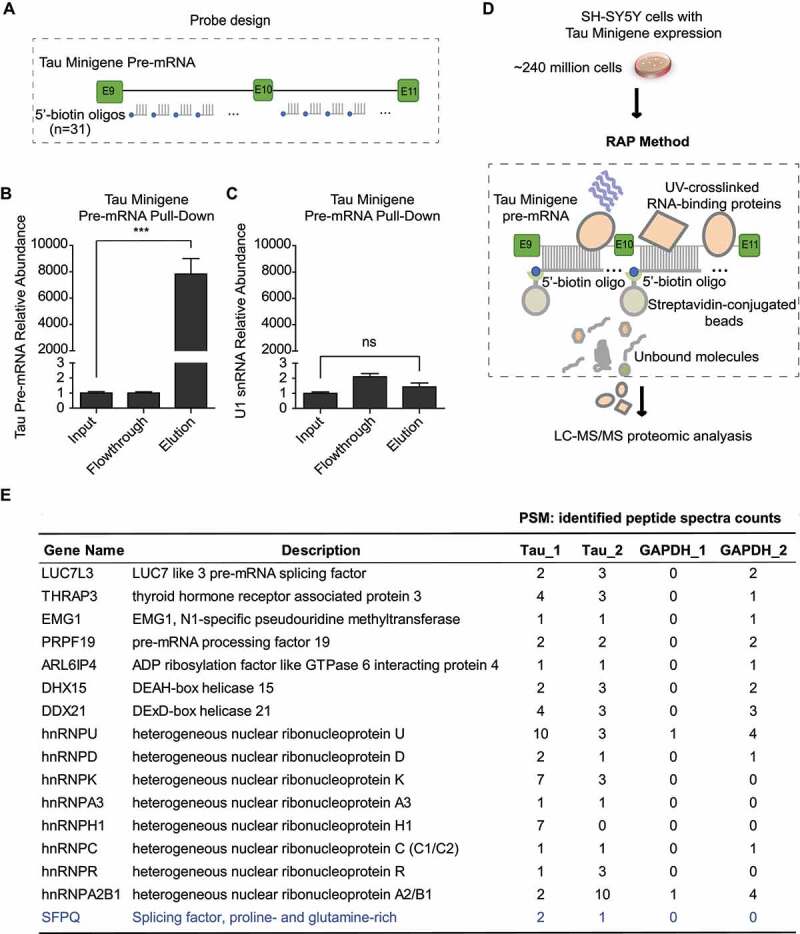
Identification of proteins binding to the Tau minigene pre-mRNA using the RAP- MS method. (a) 5ʹ biotin labelled 90nt DNA oligos that are complimentary to Tau minigene pre- mRNA sequence were designed as shown in the diagram. (b) The Tau minigene pre-mRNA abundance through RAP process was measured by qRT-PCR using the primers Fp and Rp as shown in Figure 1(c). (c) The U1 snRNA abundance was measured by qRT-PCR with the same samples as (b). GAPDH was used as the internal standard in both (b) and (c). Data shown in (b) and (c) represent the means ± SEM (n = 3, ***P < 0.001; ns: not significant, two-tailed Student’s t test). (d) Identification of Tau exon 10 splicing regulator candidates by performing the RAP enrichment procedure in SH-SY5Y cells that stably express the Tau minigene cassette, followed by proteomics. (e) Identified RBPs that are selectively enriched with Tau minigene probes (Tau_1 and Tau_2 are biological replicates), along with identified peptide spectra counts found by mass spec analysis, are listed. RAP-MS runs using biotinylated probes that target GAPDH mature transcript were included as controls. Known Tau exon 10 splicing regulator (SFPQ) is highlighted in blue.

Among the identified RBPs, we applied a set of criteria to select proteins that are specific to Tau minigene pre-mRNA rather than GAPDH mature transcript. These criteria are: firstly, selected proteins should present in both biological replicate of the Tau RAP-MS runs; secondly, the sum of the number of peptide spectrum matches (PSM) for each selected protein in the two Tau RAP-MS runs are at least two-fold higher compared to that in the two GAPDH negative control runs; last but not the least, in some exceptional cases, RBPs with no PSM counts in both GAPDH negative control runs and very high PSM counts (higher than 5) in one of the two Tau RAP-MS runs were also selected. Based on the above criteria, we focused on the 15 novel RBPs specific to Tau minigene pre-mRNA, as showed in Figure 2(e).

### Ribonucleoprotein immunoprecipitation (RIP) analysis

RIP assay was performed in SH-SY5Y cells with hnRNPC antibody (Abcam, USA) or IgG control (Millipore, Etobicoke, ON). 10 million SH-SY5Y cells were lysed with RIP lysis buffer (50 mM Tris-HCl pH 7.5, 100 mM NaCl, 1% NP-40, 0.1% SDS, 0.5% Sodium Deoxycholate, 1x protease inhibitor cocktail). After sonication, cell lysate was precleared with protein G beads for half an hour in 4°C. Meanwhile, 5 μg antibody was incubated with protein G beads for 1 hr at room temperature. Next, precleared cell lysate was incubated with antibody coupled protein G beads for overnight at 4°C. After 9 times of wash with NET-2 buffer (5 mM Tris-HCl pH 7.5, 150 mM NaCl, 0.1% TritonX-100), RNA was isolated with Trizol-LS (Thermo Scientific, USA) and was then reverse transcribed and measured by qRT-PCR, with 18S rRNA as the internal control. Primers are shown in Table S1.

### In vitro transcription

DNA fragments were *in vitro* transcribed into RNA using the MEGAscript T7 transcription kit (Thermo Scientific, USA) incorporated with biotinylated-CTP (Thermo Scientific, USA) at a molar ratio of 1:1 with non-biotinylated CTP. RNA products were visualized in 6% or 10% TBE-Urea gel (Thermo Scientific, USA) using SYBR Gold (Thermo Scientific, USA).

### In vitro RNA pull-down

For each reaction, 10 pmol of each *in vitro* transcribed RNA and 1.5 mg of SH-SY5Y cell lysate (in RIP lysis buffer) were used. The transcribed RNA was first incubated at 65°C for 10 mins, then cooling down slowly to 4°C. The pre-cleared cell lysate was then incubated with the RNA at 4°C for 4 hrs. After streptavidin beads capture and four washes with NET-2 buffer, proteins were eluted with the 1x LDS loading buffer and were probed with specific antibody by Western blot. Samples with beads-only and human GAPDH mRNA 3ʹUTR fragment pull-down were used as negative controls.

### RNA-Seq data processing

PSP RNA-seq dataset was obtained from Mayo RNAseq Study [38] (Synapse:syn5049298). The post-mortem tissues were collected from cerebellum region of North American Caucasian subjects with neuropathological diagnosis of PSP (n = 84) or elderly controls without neurodegeneration diagnoses (n = 77). Within this cohort, all PSP subjects were obtained from the Mayo Clinic Brain Bank (MCBB), whereas control subjects were obtained from both the MCBB and the Banner Sun Health Research Institute (Banner).

Raw counts from above RNA-seq dataset were processed and analysed by limma [39], an R/Bioconductor software package, to identify differentially expressed genes in R studio (shown in Tables S3-4). P-value was adjusted with Benjamini–Hochberg method for multiple testing. Genes with adjusted P-value < 0.05 were considered as differentially expressed.

## Results

### RAP-MS screen identifies novel Tau exon 10 splicing regulators

To validate the RAP method, we first designed biotinylated DNA probes targeting U1 snRNA and GAPDH mRNA transcripts in HEK 293 FT cells to perform the RAP RNA pull-down workflow (Figure S1A). As shown in Figure S1B-D, we confirmed the effectiveness and specificity of targeted RNA enrichment using the RAP method. Next, we worked on adapting the RAP-MS method to identify RBPs upstream of Tau exon 10 splicing in the SH-SY5Y neuroblastoma cells. Towards this end, we first confirmed that the SH-SY5Y cells express both 3 R-Tau (skipping the exon 10) and 4 R-Tau (including the exon 10) isoforms (Figure 1(b)). We optimized the UV irradiation crosslinking dose at 600 mJ/cm2 in SH-SY5Y cells (Figure S2). To identify the protein candidates that selectively bind to the Tau pre-mRNA transcript around its exon 10 region, we generated a Tau minigene cassette that includes full-length exons 9, 10, 11, flanked by the partial sequences (first and last ~1kb each) of intron 9 and intron 10 (~ 4.5 kb fragment in total) (Figure 1(c)). The exon 10 alternative splicing profile of the Tau minigene transcript is similar to that of the endogenous Tau transcript in the SH-SY5Y cells (Figure 1(c)). Once we generated an SH-SY5Y cell line that stably expressed the Tau minigene, we found the Tau minigene pre-mRNA level was ~70 fold higher than the endogenous Tau pre-mRNA level (Figure 1(d)), which would benefit the sensitivity of our RAP-MS experiment.

To capture the Tau minigene pre-mRNA, we used thirty-one 90-mer biotinylated oligo probes (Figure 2(a), Table S2) that tile the entire length of the Tau minigene sequence (non-specific and exon-targeting probes were removed). With these probes, Tau minigene pre-mRNA, but not the non-specific U1 snRNA, was highly enriched using the RAP procedure (Figure 2(b-c)). We then performed RAP-MS experiment (Figure 2(d)) with ~240 million SH-SY5Y cells expressing the Tau minigene using oligo sets that target either the Tau minigene pre-mRNA or GAPDH mature mRNA (negative control). From two RAP-MS runs with the Tau-targeting probes, we identified 66 RBPs, including 12 previously reported Tau exon 10 splicing regulators [23,26,27,40–43] (Table S5). When we compared these results to proteins that were also identified in the control RAP-MS runs using GAPDH-targeting probes, we narrowed our results down to 16 RBPs that specifically interact with the Tau minigene pre-mRNA (Figure 2(e)). Among these RBP candidates, only one (SFPQ) was previously reported to regulate Tau exon 10 splicing [27].

### Loss of function assays validate Tau exon 10 splicing regulators

**Figure 3.**
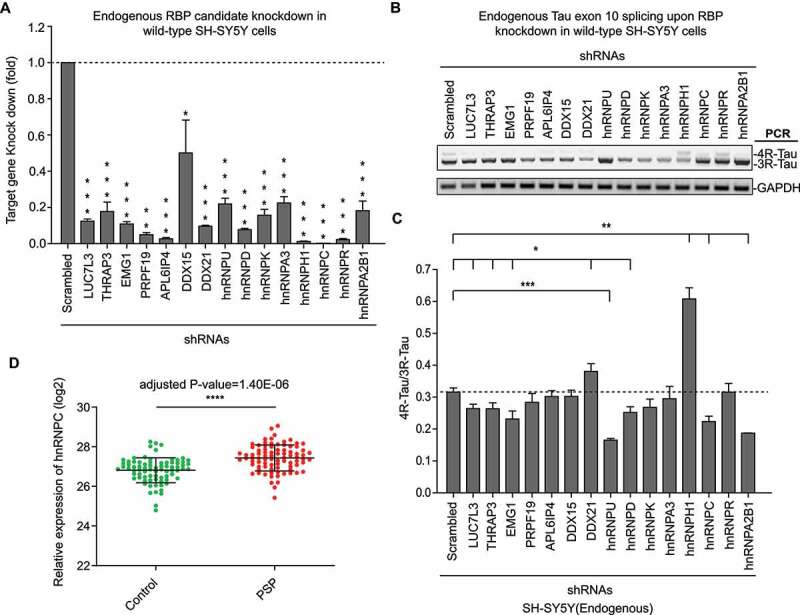
Loss-of-function assays to validate RAP-MS hits that control Tau exon 10 splicing.

To test the function of the 15 newly identified RBPs on Tau exon 10 splicing regulation, we knocked down each candidate gene using short-hairpin RNAs (shRNAs) in wild-type SH-SY5Y cells (Figure 3(a)). For 9 of these 15 candidates (i.e. LUC7L3, THRAP3, EMG1, DDX21, hnRNPD, hnRNPU, hnRNPH1, hnRNPC and hnRNPA2B1), their individual knockdown significantly (p < 0.05) impacted endogenous Tau exon 10 splicing (Figure 3(b-c)).

(a) Confirmation of knockdown of the RAP-MS hits using the hit-targeting shRNA infection groups compared with the scrambled shRNA infection group in SH-SY5Y cells, as assessed by qRT-PCR. Data shown represent the means ± SEM (n = 3, *P < 0.05; **P < 0.01; ***P < 0.001, two-tailed Student’s t test). (b-c) Measurement of endogenous Tau exon 10 splicing affected by knockdown of the RAP-MS hits in wild-type SH-SY5Y cells, as assessed by RT-PCR with the primers F and R as shown in Figure 1(b). GAPDH was used as the internal standard; each lane represents a different cell population in which a given RAP-MS hit was knocked down using shRNA; control cells transduced with scrambled shRNA were included (b). The levels of ratio (4 R-Tau relative to 3 R-Tau bands) from the endogenous MAPT gene were calculated (c). Data shown represent the means ± SEM (n = 3, *P < 0.05; **P < 0.01; ***P < 0.001, two-tailed Student’s t test). (d) RNA-seq results for Control (n = 77) versus PSP (n = 84) cerebellum released from the Mayo clinic (see method). hnRNPC expression levels were significantly increased in PSP compared to Control (****adjusted p-value < 0.0001; Benjamini-Hochberg correction).

PSP is one form of sporadic 4 R-tauopathy. To date there has been no known genetic cause has been linked. Therefore, in our study, we decided to identify novel Tau splicing factors whose dysregulation may contribute to elevated 4 R-Tau expression and subsequent 4 R-Tau aggregation in PSP. For this purpose, we re-analysed large-scale RNA-seq data of post-mortem PSP patient brain samples released by Mayo Clinic [38] (Synapse:syn5049298). Among the most significant 4 R-Tau splicing factor candidates (p < 0.01) from our RAP-MS experiments, the hnRNPC transcript is also significantly elevated (adjusted p < 0.0001) in PSP patient post-mortem brain samples versus sex- and age-matched controls (Figure 3(d)). This result suggests that elevated hnRNPC level might associate with 4 R-Tau aggregation in PSP. Next, we decided to focus on the function of hnRNPC to investigate its regulatory mechanism as a novel Tau splicing factor.

### HnRNPC interacts with Tau pre-mRNA and promote exon 10 inclusion

**Figure 4.**
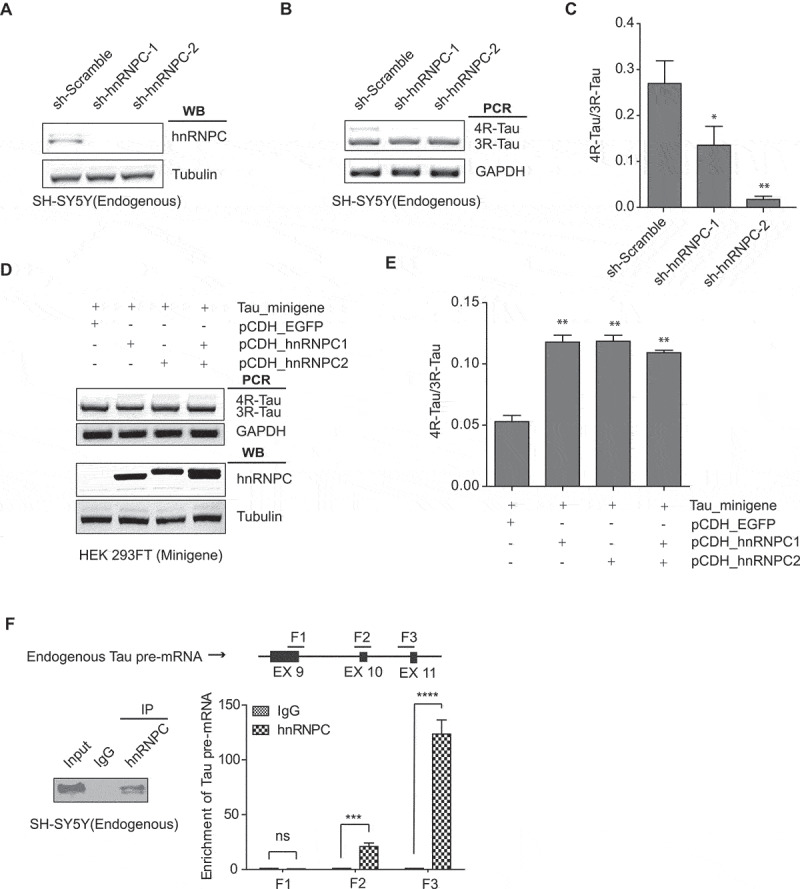
HnRNPC interacts with Tau pre-mRNA and promotes Tau exon 10 inclusion. (a) SH-SY5Y cells were transduced with lentivirus expressing two independent shRNAs targeting hnRNPC. Knockdown of hnRNPC was confirmed by Western blot analysis, with the Tubulin level as the loading control. (b-c) RT-PCR products from the endogenous Tau exon 10 splicing after hnRNPC knockdown (b). The 4 R/3 R-Tau ratios were calculated (c). Data shown represent the means ± SEM (n = 3, *P < 0.05; **P < 0.01, two-tailed Student’s t test). (d-e) Overexpression of the hnRNPC1 isoform, the hnRNPC2 isoform, or the two isoforms together all promoted Tau minigene exon 10 inclusion in HEK 293 FT cells (d). The 4 R/3 R-Tau ratios from the Tau minigene were calculated (e). Data shown represent the means ± SEM (n = 3, **P < 0.01, two-tailed Student’s t test). (f) RIP analysis to measure the enrichment of endogenous Tau pre-mRNA in hnRNPC IP relative to IgG IP as measured by qRT-PCR using the primers target F1, F2 and F3 in wild-type SH-SY5Y cells, 18s rRNA was used as internal control. Data shown represent the means ± SEM (n = 3, ***P < 0.001; ****P < 0.0001; ns: not significant, two-tailed Student’s t test).

To confirm the functional role of hnRNPC in Tau exon 10 splicing regulation, we induced hnRNPC knockdown using two independent shRNAs in SH-SY5Y cells and found that 4 R-/3 R-Tau ratio was significantly decreased (Figure 4(a-c)). Two hnRNPC splice isoforms, namely hnRNPC1 and hnRNPC2, form tetramers at the molar ratio of 3:1. These tetramers then further form higher-ordered trimers to occupy ~700 nucleotides for alternative splicing regulation [44]. We demonstrated that overexpression of either of these two hnRNPC isoforms can promote inclusion of Tau exon 10 in the minigene in HEK 293 FT cells (Figure 4(d-e)) and in HeLa cells (Figure S3). Given earlier evidence generated by our RAP-MS experiments showing that hnRNPC associated with Tau pre-mRNA, we performed ribonucleoprotein immunoprecipitation (RIP) assay to validate this protein-RNA interaction. After we immunoprecipitated hnRNPC in wild-type SH-SY5Y cell extracts, we measured endogenous Tau pre-mRNA using three sets of primers (Figure 4(f)). The interaction was quantified by the enrichment of Tau pre-mRNA in hnRNPC immunoprecipitation (IP) samples compared to immunoglobulin G (IgG) IP controls (Figure 4(f)). RIP results indicate that hnRNPC interacts with Tau pre-mRNA, especially at the regions near exon 10 and exon 11.

### HnRNPC promotes Tau exon 10 splicing through direct binding to U-tracts around exon 10

**Figure 5.**
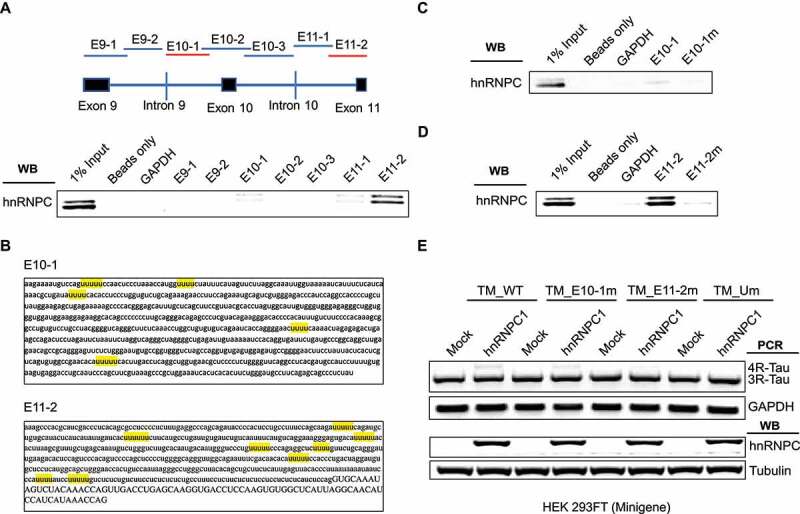
HnRNPC promotes Tau exon 10 inclusion through direct binding to U-rich tracts near Tau exons 10 and 11. (a) hnRNPC interacts with Tau pre-mRNA at the boundary between Intron 10 and Exon 11 and Intron 9 flanking Exon 10, as detected by *in vitro* RNA pull-down assay. *In vitro* transcribed RNA fragments (with biotin tag) covering the indicated regions (top diagram) around Tau pre-mRNA exons 9,10 and 11 were incubated with wild-type SH-SY5Y cell lysate. RNA fragments were then isolated using streptavidin resin. The resulted protein eluents were probed with antibody for hnRNPC. (b) RNA sequences for E10-1 and E11-2. Red indicates binding regions of hnRNPC in Tau minigene pre-mRNA. U-tract (at least 4 uridines in a row) positions are highlighted in yellow. Lowercase letters indicate intronic sequence, capital letters indicate exonic sequence. (c) Interaction between hnRNPC and Tau pre-mRNA E10-1 fragment is abolished after point mutations disrupting all U-tracts (by replacing the middle uridine with cytosine in each U-tract) in the fragment sequence, as measured by *in vitro* RNA pull-down assay. E10-1 m represents the E10-1 fragment with all U-tracts mutated as described above. (d) Interaction between hnRNPC and Tau pre-mRNA E11-2 fragment is abolished after point mutations disrupting all U-tracts (shown as E11-2 m). (e) The impact of hnRNPC on Tau exon 10 inclusion was partially abolished when co-expressed with Tau minigene containing U-tract mutations in either E10-1 or E11-2 (shown as TM_E10-1 m and TM_E11-2 m separately). The hnRNPC-induced Tau exon 10 inclusion was completely abolished when co-expressed with Tau minigene containing U-tract mutations in both of these fragments (shown as TM_Um). TM_WT represents the wild-type Tau minigene. Results shown are from three independent experiments.

To further define hnRNPC binding regions around exon 10 in the Tau pre-mRNA, we performed *in vitro* RNA pull-down in wild-type SH-SY5Y cell extracts with the seven *in vitro* synthesized RNA fragments (~700 nt with 150 nt overlap with each other) from the Tau minigene sequence (Figure 5(a)). Pull-down analysis showed that hnRNPC was associated with the E10-1, E11-1, and E11-2 fragments (Figure 5(a), S4). As reported in previous studies, hnRNPC has a conserved binding motif with a poly-uridine sequence or U-tract [45]. Our pull-down analysis showed that hnRNPC was associated with the E10-1 and E11-2 fragments (Figure 5(a), S4), both of which have multiple U-tracts binding sites (Figure 5(b)). We also noticed that hnRNPC weakly associated with the E11-1 fragment. However, when we mutated the two U-tracts that overlapped between E11-1 and E11-2 by replacing the middle uridine with cytosine, the binding between hnRNPC and E11-1 was abolished (Figure S5), suggesting that this weak interaction is resulted from the U-tracts overlapping between E11-1 and E11-2. To further study the functional binding sites for hnRNPC around the Tau exon 10 region, we mutated all U-tracts found in the E10-1 and E11-2 fragments separately, through replacing the middle uridine with cytosine as described above. As a result, we found that the binding affinity of hnRNPC to either of these two fragments was totally abolished with the mutations (Figure 5(c-d), S6-7).

To gain further insight into the specific hnRNPC RNA binding motifs for Tau pre-mRNA regions, we further made constructs of Tau minigene with mutated U-tracts in the E10-1, E11-2, or both regions. We then co-transfected these mutated Tau minigene constructs with hnRNPC in HEK 293 FT cells. As shown (Figure 5(e)), overexpression of hnRNPC with wild-type Tau minigene in HEK 293 FT cells significantly promoted Tau minigene exon 10 inclusion. In comparison, the impact of hnRNPC overexpression on Tau minigene exon 10 inclusion was fully blocked in Tau minigene with U-tract mutations in the E10-1 and E11-2 regions. These results demonstrate that U-tracts within the E10-1 and E11-2 regions can be both recognized and utilized by hnRNPC to promote Tau exon 10 inclusion.

**Figure 6.**
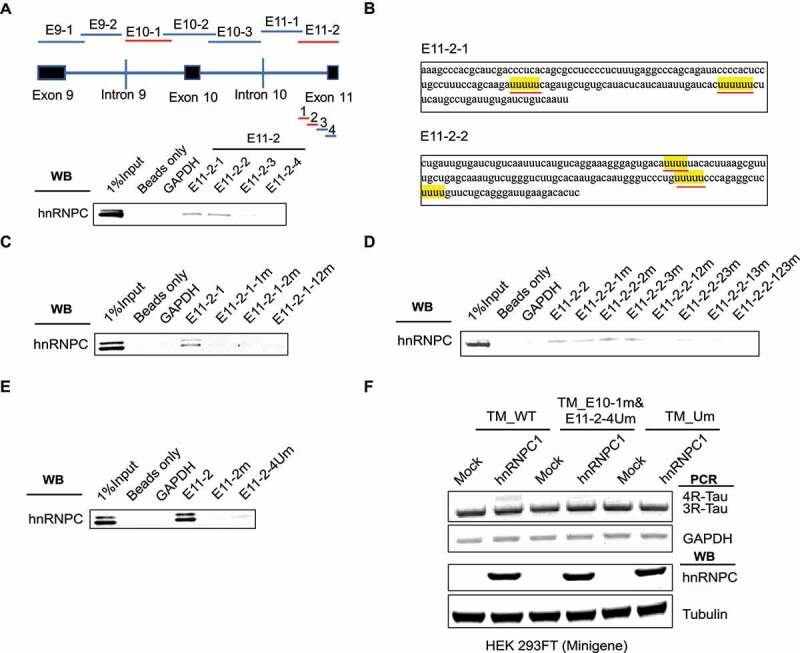
Detailed characterization of hnRNPC binding sites near Tau exon 11. (a) E11-2 is divided into four sub-fragments, 150 nt each with 20 nt overlap. hnRNPC interacts with E11-2-1 and E11-2-2 as detected by *in vitro* RNA pull-down assay. The binding fragments are marked in red. (b) U-tract positions in E11-2-1 and E11-2-2 are highlighted in yellow. (c) Interaction between hnRNPC and the E11-2-1 sub-fragment with mutated U-tract motifs (by replacing the middle uridine with cytosine) separately and altogether. E11-2-1-1 m represents first U-tract was mutated; E11-2-1-2 m represents second U-tract was mutated; E11-2-1-12 m represents both U- tracts were mutated. (d) Interaction between hnRNPC and the E11-2-2 sub-fragment with mutated U-tract motifs following the same procedure described above. (e) Interaction between hnRNPC and E11-2 fragment is largely abolished with the mutations in first four U-tracts (shown as E11- 2-4Um) compared to mutations in all U-tracts. (f) First four U-tracts in E11-2 fragment (underlined in red in panel b) together with the U-tracts in E10-1 fragment were mutated in Tau minigene (shown as TM_E10-1 m&E11-2-4Um). The impact of hnRNPC on Tau exon 10 inclusion was mostly abolished when co-expressed with this mutated Tau minigene compared to full U- tracts mutated Tau minigene that described in Figure 5(e). Results shown are from three independent experiments.

To further narrow down the functional binding sites for hnRNPC in the E11-2 region, we divided E11-2 into four sub-fragments (150 nt with 20-bp overlap with each other) as shown (Figure 6(a)). Next, we performed *in vitro* RNA pull-down and found that hnRNPC was predominantly associated with the first two out of the four tested sub-fragments within the E11-2 region (Figure 6(a), S8). Through combined mutation for U-tracts within these two sub-fragments (Figure 6(b)), we found that the first four U-tracts (within E11-2-1 and E11-2-2 sub-fragments) are necessary for interaction with hnRNPC (Figure 6(c-d), S9-10). We then mutated the first four U-tracts in the E11-2 fragment and showed that the hnRNPC binding affinity was mostly abolished, which is similar to the phenotype when all U-tracts were mutated in the E11-2 fragment (Figure 6(e), S11). On the other hand, since the binding of hnRNPC to the E10-1 region was weak, we were not able to further narrow down hnRNPC binding sites within this region through *in vitro* RNA pull-down experiments.

Importantly, we showed that Tau minigene exon 10 inclusion promoted by hnRNPC overexpression was largely abolished after the five U-tracts in the E10-1 region and the first four U-tracts in the E11-2 region were all mutated in the Tau minigene (Figure 6(f)). These results mostly recapitulated the Tau minigene exon 10 splicing phenotypes after all U-tracts in the E10-1 and E11-2 regions were mutated (Figure 5(e)). Hence, the first four U-tracts in the E11-2 region together with the five U-tracts in the E10-1 region collectively play a crucial role for hnRNPC to bind to Tau pre-mRNA and to promote Tau exon 10 inclusion.

## Discussion

In this study, we identified novel RBPs that contribute to alternative splicing of Tau exon 10 in SY-SY5Y cells by using RAP-MS. With this approach, RBPs were pre-covalently linked to Tau pre-mRNA with UV irradiation, and were then captured by *in vitro* synthesized biotin-labelled Tau probes that were complimentary to the sequence of Tau pre-mRNA. In total, we identified fifteen novel RBPs that associate with Tau pre-mRNA and nine (i.e. LUC7L3, THRAP3, EMG1, DDX21, hnRNPD, hnRNPU, hnRNPH1, hnRNPC and hnRNPA2B1) were found to play a role in regulating Tau exon 10 splicing. Specifically, we found the hnRNPC that we validated to promote 4 R-Tau expression is also upregulated in PSP at the transcript level, a sporadic 4 R-Tauopathy, which suggests the putative correlation between hnRNPC expression and disease progression of PSP. As a note of caution, we also wanted to point out that transcript and protein levels of a gene may not necessarily correlate in the same samples.

HnRNPC is well known to regulate RNA processing and pre-mRNA splicing [46]. Previous studies reported that hnRNPC might play a role in cancer progression, such as breast cancer and colon cancer [47–49]. However, limited efforts have been made to unveil the functional role of hnRNPC in nervous system. In this study, we found that hnRNPC physically interacts with Tau pre-mRNA around its exon 10 through the predicted U-tract binding motif [45] to promote Tau exon 10 inclusion. Several regulatory elements including exonic and intronic enhancers and silencers have been characterized within Tau exon 10 and its 5ʹ splice site (5’ss) present at the exon 10-intron 10 junction [50]. Previous work has also predicted that the exon-intron junctions at the 3ʹend of exon 10 forms a stem loop structure and plays an essential role in Tau exon 10 splicing [21]. Many FTDP-related mutations have been identified in this stem loop region, which may destabilize the specific structure for splicing factor binding [18]. Conversely, in our study, we found that the hnRNPC-binding U-tracts around the 5ʹ-end of both exons 10 and 11, rather than inside of exon 10 or the aforementioned stem loop region at the 3ʹ-end of exon 10, are more important for hnRNPC to direct Tau exon 10 inclusion. Our findings provide insights for novel molecular mechanisms of hnRNPC that direct Tau exon 10 splicing, which may compensate the existing knowledge. In addition, the upregulation of hnRNPC in the population of PSP patients suggests the potential therapeutic role of hnRNPC in alleviating the disease progression of PSP. Moreover, modulating the expression of hnRNPC might be another approach to restore the excessive expression of 4 R-Tau in FDTP-17 patients that bearing the silence and intronic mutations around exon 10.

Lack of robust approaches to study upstream splicing regulators has greatly restricted the research progress of splicing field. Even though *in vitro* RNA pull-down based proteomic approaches have been widely used to detect locus specific RBPs, the high false positive rate induced by prior cell lysis, re-establishment of RNA-protein interaction and gentle purification conditions have markedly increased the work burden for subsequent validation. Instead of using *in vitro* transcribed RNA to pull down proteins that released by cell lysis, the recent developed RAP-MS fixed the RNA protein interaction in situ and used a groups of DNA probes to pull down RNA along with its covalently conjugated RBPs, which is available to detect the RNA-protein interaction in native state. Moreover, in denatured conditions during hybridization and purification steps, RAP-MS can be utilized to identify proteins that directly bind to RNA of interests. RAP-MS was initially developed to identify the RBPs that function to regulate lncRNA Xist. In this study, it is the first time that this method been applied to splicing field. Compared to most RNA splicing regulator studies that depend on *in vitro* RNA fragment pull-down in the context of cell or tissue lysate [33], the RAP-MS method allows for rapid narrowing-down and identification of novel regulators upstream of specific splicing events with high positive rate. It is also a much more directed and efficient way to identify splicing regulators upstream of specific splicing events than large-scale RBP eCLIP-seq analysis and loss-of-function screens that are currently available to the splicing research community.

In this study, one technical limitation of our approach is that we still needed to exogenously express the Tau minigene to identify regulators that specifically mediate Tau exon 10 splicing. Although we were able to efficiently capture endogenous Tau pre-mRNA in addition to the Tau minigene pre-mRNA (Figure S12), to directly identify RBPs that interact with the endogenous Tau pre-mRNA around its exon 10, we will need to develop a new procedure to selectively isolate and enrich for the endogenous Tau pre-mRNA sequence in that region. With this future improvement, the RAP-MS method can be applied more widely in the fields of RNA splicing and other RNA regulation mechanisms. For example, in the context of Tauopathies, RAP-MS with the endogenous Tau pre-mRNA can be applied to directly unveil the deregulated RBPs that natively interact with Tau pre-mRNA in post-mortem brain tissues collected from tauopathy patients.

Altogether, in this study, we identified novel Tau splicing regulators using RAP-MS. Among the identified RBP hits, we further revealed the regulatory mechanisms of hnRNPC as a novel splicing factor to promote inclusion of Tau exon 10. In addition, our successful application of RAP-MS method in identification of Tau splicing factors indicates that RAP-MS can be routinely used as a sensitive and comprehensive tool to unveil *in situ* splicing factors that mediate aberrant splicing events clinically linked with human diseases such as cancer and neurodegeneration.

## Supplementary Material

Supplemental Material

Supplemental Material

## Data Availability

All data generated or analyzed during this study are included in the article. The mass spectrometry proteomics data have been deposited to the ProteomeXchange Consortium via the PRIDE partner repository with the dataset identifier PXD023523.

## References

[1] Kovacs GG. Invited review: neuropathology of tauopathies: principles and practice. Neuropathol Appl Neurobiol. 2015;41:3–23. 25495175 10.1111/nan.12208

[2] Barbier P, Zejneli O, Martinho M, et al. Role of Tau as a microtubule-associated protein: structural and functional aspects. Front Aging Neurosci. 2019;11:204. 31447664 10.3389/fnagi.2019.00204 PMC6692637

[3] Spillantini MG, Goedert M. Tau pathology and neurodegeneration. Lancet Neurol. 2013;12:609–622. 23684085 10.1016/S1474-4422(13)70090-5

[4] Wang Y, Mandelkow E. Tau in physiology and pathology. Nat Rev Neurosci. 2016;17:5–21. 26631930 10.1038/nrn.2015.1

[5] Caillet-Boudin ML, Fernandez-Gomez FJ, Tran H, et al. Brain pathology in myotonic dystrophy: when tauopathy meets spliceopathy and RNAopathy. Front Mol Neurosci. 2014;6:57. 24409116 10.3389/fnmol.2013.00057 PMC3885824

[6] Crary JF, Trojanowski JQ, Schneider JA, et al. Primary age-related tauopathy (PART): a common pathology associated with human aging. Acta Neuropathol. 2014;128:755–766. 25348064 10.1007/s00401-014-1349-0 PMC4257842

[7] Sergeant N, Delacourte A, Buee L. Tau protein as a differential biomarker of tauopathies. Biochim Biophys Acta. 2005;1739:179–197. 15615637 10.1016/j.bbadis.2004.06.020

[8] Avila J. Tau phosphorylation and aggregation in Alzheimer’s disease pathology. FEBS Lett. 2006;580:2922–2927. 16529745 10.1016/j.febslet.2006.02.067

[9] Buee L, Troquier L, Burnouf S, et al. From tau phosphorylation to tau aggregation: what about neuronal death? Biochem Soc Trans. 2010;38:967–972. 20658986 10.1042/BST0380967

[10] Hong Y, Chan CB, Kwon IS, et al. SRPK2 phosphorylates tau and mediates the cognitive defects in Alzheimer’s disease. J Neurosci. 2012;32:17262–17272. 23197718 10.1523/JNEUROSCI.3300-12.2012 PMC3518045

[11] Stoothoff WH, Johnson GV. Tau phosphorylation: physiological and pathological consequences. Biochim Biophys Acta. 2005;1739:280–297. 15615646 10.1016/j.bbadis.2004.06.017

[12] Morris M, Knudsen GM, Maeda S, et al. Tau post-translational modifications in wild-type and human amyloid precursor protein transgenic mice. Nat Neurosci. 2015;18:1183–1189. 26192747 10.1038/nn.4067 PMC8049446

[13] Iqbal K, Gong CX, Liu F. Hyperphosphorylation-induced tau oligomers. Front Neurol. 2013;4:112. 23966973 10.3389/fneur.2013.00112 PMC3744035

[14] Goedert M, Spillantini MG, Jakes R, et al. Multiple isoforms of human microtubule-associated protein tau: sequences and localization in neurofibrillary tangles of Alzheimer’s disease. Neuron. 1989;3:519–526. 2484340 10.1016/0896-6273(89)90210-9

[15] Hefti MM, Farrell K, Kim S, et al. High-resolution temporal and regional mapping of MAPT expression and splicing in human brain development. PLoS One. 2018;13:e0195771. 29634760 10.1371/journal.pone.0195771 PMC5892924

[16] Goedert M, Spillantini MG, Potier MC, et al. Cloning and sequencing of the cDNA encoding an isoform of microtubule-associated protein tau containing four tandem repeats: differential expression of tau protein mRNAs in human brain. EMBO J. 1989;8:393–399. 2498079 10.1002/j.1460-2075.1989.tb03390.x PMC400819

[17] Kar A, Kuo D, He R, et al. Tau alternative splicing and frontotemporal dementia. Alzheimer Dis Assoc Disord. 2005;19(Suppl. 1):S29–36. 16317255 10.1097/01.wad.0000183082.76820.81 PMC2001171

[18] Hong M, Zhukareva V, Vogelsberg-Ragaglia V, et al. Mutation-specific functional impairments in distinct tau isoforms of hereditary FTDP-17. Science. 1998;282:1914–1917. 9836646 10.1126/science.282.5395.1914

[19] Hutton M, Lendon CL, Rizzu P, et al. Association of missense and 5ʹ-splice-site mutations in tau with the inherited dementia FTDP-17. Nature. 1998;393:702–705. 9641683 10.1038/31508

[20] Lamb R, Rohrer JD, Lees AJ, et al. Progressive supranuclear palsy and corticobasal degeneration: pathophysiology and treatment options. Curr Treat Options Neurol. 2016;18:42. 27526039 10.1007/s11940-016-0422-5 PMC4985534

[21] Liu F, Gong CX. Tau exon 10 alternative splicing and tauopathies. Mol Neurodegener. 2008;3:8. 18616804 10.1186/1750-1326-3-8 PMC2483273

[22] Qian W, Liu F. Regulation of alternative splicing of tau exon 10. Neurosci Bull. 2014;30:367–377. 24627328 10.1007/s12264-013-1411-2 PMC5562657

[23] Qian W, Liang H, Shi J, et al. Regulation of the alternative splicing of tau exon 10 by SC35 and Dyrk1A. Nucleic Acids Res. 2011;39:6161–6171. 21470964 10.1093/nar/gkr195 PMC3152345

[24] Jiang Z, Tang H, Havlioglu N, et al. Mutations in tau gene exon 10 associated with FTDP-17 alter the activity of an exonic splicing enhancer to interact with Tra2 beta. J Biol Chem. 2003;278:18997–19007. 12649279 10.1074/jbc.M301800200 PMC2140226

[25] Kar A, Havlioglu N, Tarn WY, et al. RBM4 interacts with an intronic element and stimulates tau exon 10 inclusion. J Biol Chem. 2006;281:24479–24488. 16777844 10.1074/jbc.M603971200 PMC2072872

[26] Wu JY, Kar A, Kuo D, et al. SRp54 (SFRS11), a regulator for tau exon 10 alternative splicing identified by an expression cloning strategy. Mol Cell Biol. 2006;26:6739–6747. 16943417 10.1128/MCB.00739-06 PMC1592875

[27] Ishigaki S, Fujioka Y, Okada Y, et al. Altered Tau isoform ratio caused by loss of FUS and SFPQ function leads to FTLD-like phenotypes. Cell Rep. 2017;18:1118–1131. 28147269 10.1016/j.celrep.2017.01.013

[28] Gu J, Chen F, Iqbal K, et al. Transactive response DNA-binding protein 43 (TDP-43) regulates alternative splicing of tau exon 10: implications for the pathogenesis of tauopathies. J Biol Chem. 2017;292:10600–10612. 28487370 10.1074/jbc.M117.783498 PMC5481566

[29] Glatz DC, Rujescu D, Tang Y, et al. The alternative splicing of tau exon 10 and its regulatory proteins CLK2 and TRA2-BETA1 changes in sporadic Alzheimer’s disease. J Neurochem. 2006;96:635–644. 16371011 10.1111/j.1471-4159.2005.03552.x

[30] Wang Y, Wang J, Gao L, et al. Tau exons 2 and 10, which are misregulated in neurodegenerative diseases, are partly regulated by silencers which bind a SRp30c.SRp55 complex that either recruits or antagonizes htra2beta1. J Biol Chem. 2005;280:14230–14239. 15695522 10.1074/jbc.M413846200

[31] Andreadis A. Tau gene alternative splicing: expression patterns, regulation and modulation of function in normal brain and neurodegenerative diseases. Biochim Biophys Acta. 2005;1739:91–103. 15615629 10.1016/j.bbadis.2004.08.010

[32] Ule J, Blencowe BJ. Alternative splicing regulatory networks: functions, mechanisms, and evolution. Mol Cell. 2019;76:329–345. 31626751 10.1016/j.molcel.2019.09.017

[33] Grammatikakis I, Zhang P, Panda AC, et al. Alternative splicing of neuronal differentiation factor TRF2 regulated by HNRNPH1/H2. Cell Rep. 2016;15:926–934. 27117401 10.1016/j.celrep.2016.03.080 PMC4856555

[34] Chu C, Zhang QC, Da Rocha ST, et al. Systematic discovery of Xist RNA binding proteins. Cell. 2015;161:404–416. 25843628 10.1016/j.cell.2015.03.025 PMC4425988

[35] McHugh CA, Chen CK, Chow A, et al. The Xist lncRNA interacts directly with SHARP to silence transcription through HDAC3. Nature. 2015;521:232–236. 25915022 10.1038/nature14443 PMC4516396

[36] Ye J, Coulouris G, Zaretskaya I, et al. Primer-BLAST: a tool to design target-specific primers for polymerase chain reaction. BMC Bioinformatics. 2012;13:134. 22708584 10.1186/1471-2105-13-134 PMC3412702

[37] Perez-Riverol Y, Csordas A, Bai J, et al. The PRIDE database and related tools and resources in 2019: improving support for quantification data. Nucleic Acids Res. 2019;47:D442–d450. 30395289 10.1093/nar/gky1106 PMC6323896

[38] Allen M, Carrasquillo MM, Funk C, et al. Human whole genome genotype and transcriptome data for Alzheimer’s and other neurodegenerative diseases. Sci Data. 2016;3:160089. 27727239 10.1038/sdata.2016.89 PMC5058336

[39] Ritchie ME, Phipson B, Wu D, et al. limma powers differential expression analyses for RNA-sequencing and microarray studies. Nucleic Acids Res. 2015;43:e47. 25605792 10.1093/nar/gkv007 PMC4402510

[40] Yu Q, Guo J, Zhou J. A minimal length between tau exon 10 and 11 is required for correct splicing of exon 10. J Neurochem. 2004;90:164–172. 15198676 10.1111/j.1471-4159.2004.02477.x

[41] Kondo S, Yamamoto N, Murakami T, et al. Tra2 beta, SF2/ASF and SRp30c modulate the function of an exonic splicing enhancer in exon 10 of tau pre-mRNA. Genes Cells. 2004;9:121–130. 15009090 10.1111/j.1356-9597.2004.00709.x

[42] Gao L, Wang J, Wang Y, et al. SR protein 9G8 modulates splicing of tau exon 10 via its proximal downstream intron, a clustering region for frontotemporal dementia mutations. Mol Cell Neurosci. 2007;34:48–58. 17137791 10.1016/j.mcn.2006.10.004 PMC1866282

[43] Kar A, Fushimi K, Zhou X, et al. RNA helicase p68 (DDX5) regulates tau exon 10 splicing by modulating a stem-loop structure at the 5ʹ splice site. Mol Cell Biol. 2011;31:1812–1821. 21343338 10.1128/MCB.01149-10 PMC3133221

[44] Rech JE, LeStourgeon WM, Flicker PF. Ultrastructural morphology of the hnRNP C protein tetramer. J Struct Biol. 1995;114:77–83. 7612399 10.1006/jsbi.1995.1007

[45] Zarnack K, Konig J, Tajnik M, et al. Direct competition between hnRNP C and U2AF65 protects the transcriptome from the exonization of Alu elements. Cell. 2013;152:453–466. 23374342 10.1016/j.cell.2012.12.023 PMC3629564

[46] Swanson MS, Nakagawa TY, LeVan K, et al. Primary structure of human nuclear ribonucleoprotein particle C proteins: conservation of sequence and domain structures in heterogeneous nuclear RNA, mRNA, and pre-rRNA-binding proteins. Mol Cell Biol. 1987;7:1731–1739. 3110598 10.1128/mcb.7.5.1731-1739.1987 PMC365274

[47] Huang H, Han Y, Zhang C, et al. HNRNPC as a candidate biomarker for chemoresistance in gastric cancer. Tumour Biol. 2016;37:3527–3534. 26453116 10.1007/s13277-015-4144-1

[48] Wu Y, Zhao W, Liu Y, et al. Function of HNRNPC in breast cancer cells by controlling the dsRNA-induced interferon response. *EMBO J*. 2018;37(23):e99017. 10.15252/embj.201899017 PMC6276880 30158112

[49] Fischl H, Neve J, Wang ZQ, et al. hnRNPC regulates cancer-specific alternative cleavage and polyadenylation profiles. Nucleic Acids Res. 2019;47:7580–7591. 31147722 10.1093/nar/gkz461 PMC6698646

[50] D’Souza I, Schellenberg GD. tau Exon 10 expression involves a bipartite intron 10 regulatory sequence and weak 5ʹ and 3ʹ splice sites. J Biol Chem. 2002;277:26587–26599. 12000767 10.1074/jbc.M203794200

