# Supplementary material for: Identification of HnRNPC as a novel Tau exon 10 splicing factor using RNA antisense purification mass spectrometry
Source: RNA Biol. 2021 Dec 29;19(1):104–16. doi: 10.1080/15476286.2021.2015175 (PMC8786334; doi:10.1080/15476286.2021.2015175)
Supplement: Supplemental Material [file KRNB_A_2015175_SM5577.zip › supplementary/Xing et al supp_figures highlights FOR REVIEW ONLY.docx]

**Identification of HnRNPC as A Novel Tau Exon 10 Splicing Factor Using RNA Antisense Purification Mass Spectrometry**


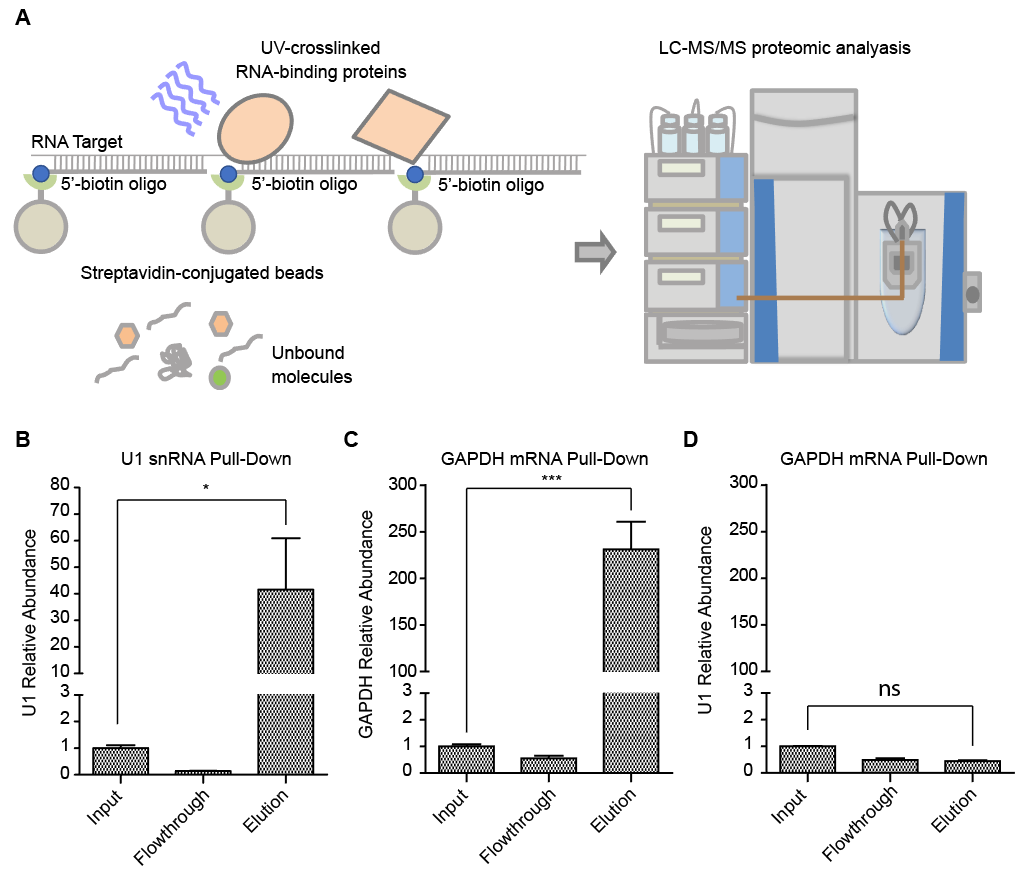


**Figure S1: RNA antisense purification (RAP) proteomic method to identify RNA-binding proteins specifically interact with RNA targets.** (A) RAP workflow. Cells are irradiated with UV light (254nm) to induce RNA-protein crosslinking. After cell lysis, target RNA molecules are hybridized with a pool of 90nt oligos with 5’-biotinylationand and are enriched together with the cross-linked RBP interaction partners by streptavidin beads. These RBPs are digested on beads and are identified using LC-MS/MS proteomics. (B) Enrichment of U1 snRNA in HEK 293FT cells using RAP method. U1 snRNA abundance was measured by qRT-PCR, with GAPDH as the internal standard. Data shown represent the means ± SEM (n = 3, *P < 0.05, two-tailed Student's t test). (C-D) Specific enrichment of GAPDH mRNA in HEK 293FT cells using the RAP method. (C) GAPDH mRNA and (D) U1 snRNA abundances were measured by qRT-PCR, with ACTB as the internal standard. Data shown represent the means ± SEM (n = 3, ***P < 0.001; ns: not significant, two-tailed Student's t test)


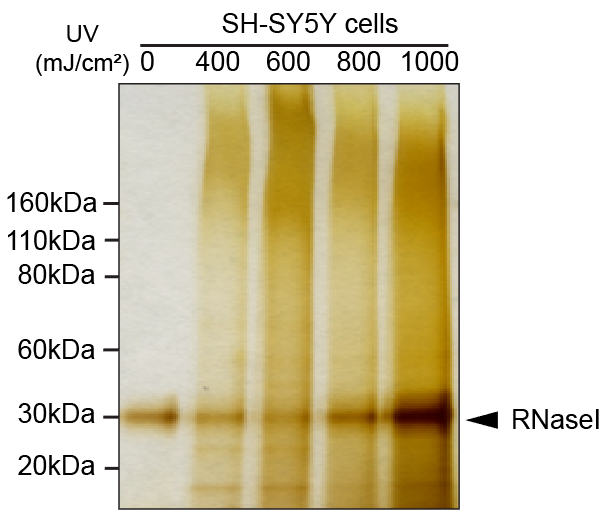


**Figure S2: UV-crosslink optimization in SH-SY5Y.** Proteins cross-linked to mRNAs with UV irradiation were captured by oligo-dT beads and released by RNase I digestion, before being loaded onto the SDS-PAGE and detected by silver staining.


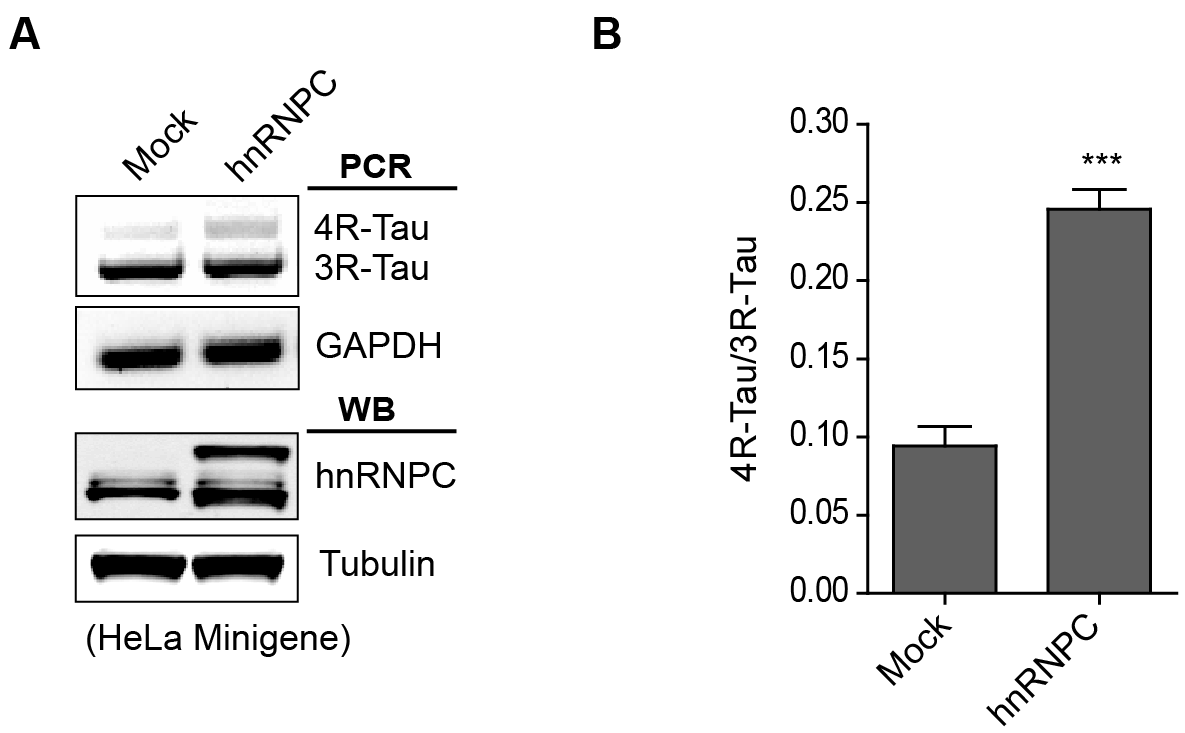


**Figure S3: Overexpression of hnRNPC promotes Tau minigene exon 10 inclusion in HeLa cells.** Forty-eight hours post-transfection, cells were harvested for Tau exon 10 splicing detection using RT-PCR. GAPDH was used as the internal control(A). The 4R/3R-Tau ratios from the Tau minigene were calculated (B). Data shown represent the means ± SEM (n = 3, ***P < 0.001, two-tailed Student's t test)


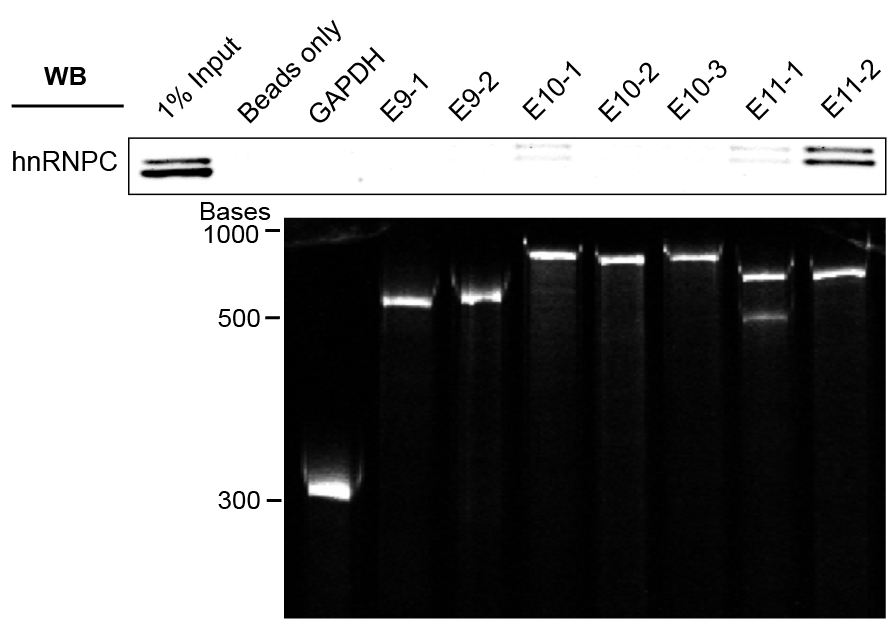


**Figure S4: Interaction between hnRNPC and the seven *in vitro* synthesized RNA fragments from the Tau minigene sequence was measured by *in vitro* RNA/protein interaction assay.** Top: The binding affinity of hnRNPC to this region was detected by *in vitro RNA* pull-down assay. Bottom: 20% Input of RNA fragments were loaded in the home-made 10% TBE-Urea gel and visualized with SYBR Gold (Thermo Scientific, USA).


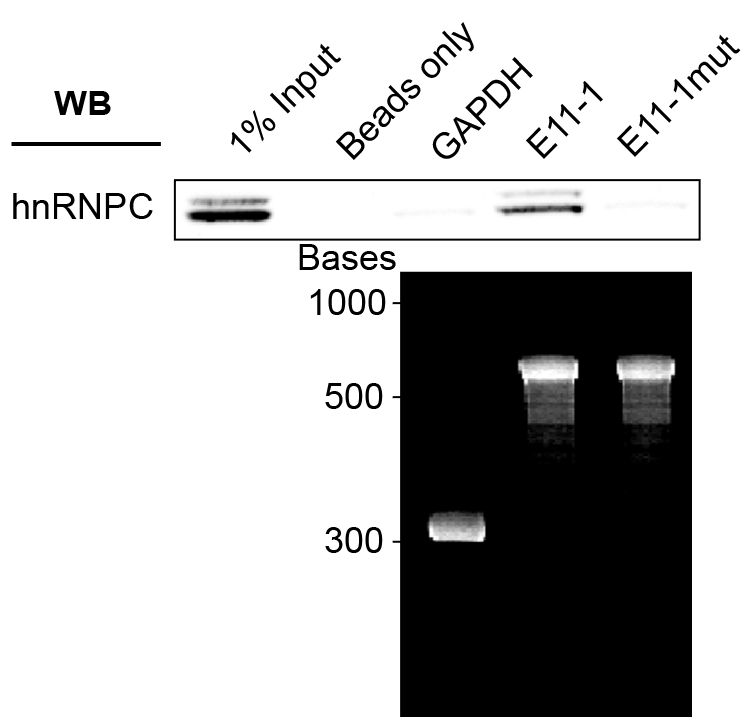


**Figure S5: Binding affinity of hnRNPC with E11-1 was disrupted after mutation of the overlapped U-tracts shown in E11-2.** Top: The binding affinity of hnRNPC to this region was detected by *in vitro RNA* pull-down assay. E11-1mut: U-tracts mutant in overlapped region. E11-1 is a positive binding control. Bottom: 20% Input of RNA fragments were loaded in the 6% TBE-Urea gel (Thermo Scientific, USA) and visualized with SYBR Gold(Thermo Scientific, USA).


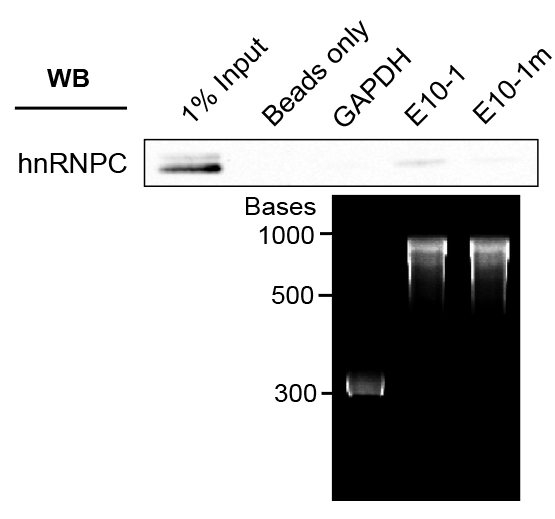


**Figure S6: Binding affinity of hnRNPC with E10-1 was disrupted after mutation of all the U-tracts shown in E10-1.** Top: The binding affinity of hnRNPC to this region was detected by *in vitro RNA* pull-down assay. E10-1m represents the E10-1 fragment with all U-tracts mutated. E10-1 is a positive binding control. Bottom: 20% Input of RNA fragments were loaded in the 6% TBE-Urea gel (Thermo Scientific, USA) and visualized with SYBR Gold(Thermo Scientific, USA).


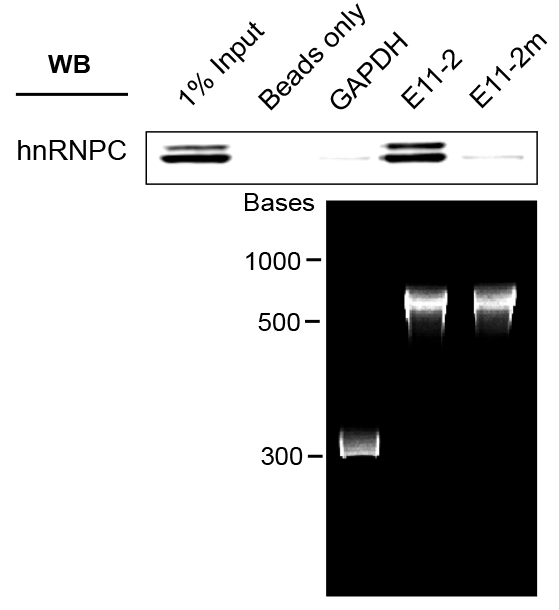


**Figure S7: Binding affinity of hnRNPC with E11-2 was disrupted after mutation of all the U-tracts shown in E11-2.** Top: The binding affinity of hnRNPC to this region was detected by *in vitro RNA* pull-down assay. E11-2m represents the E11-2 fragment with all U-tracts mutated. E11-2 is a positive binding control. Bottom: 20% Input of RNA fragments were loaded in the 6% TBE-Urea gel (Thermo Scientific, USA) and visualized with SYBR Gold (Thermo Scientific, USA).


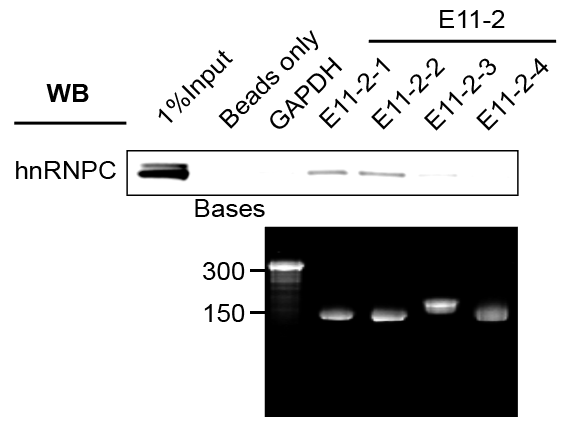


**Figure S8: Interaction between hnRNPC and the four sub-fragments from E11-2 sequence was measured by *in vitro* RNA/protein interaction assay.** Top: The binding affinity of hnRNPC to this region was detected by *in vitro RNA* pull-down assay. Bottom: 20% Input of RNA fragments were loaded in the 10% TBE-Urea gel (Thermo Scientific, USA) and visualized with SYBR Gold (Thermo Scientific, USA).


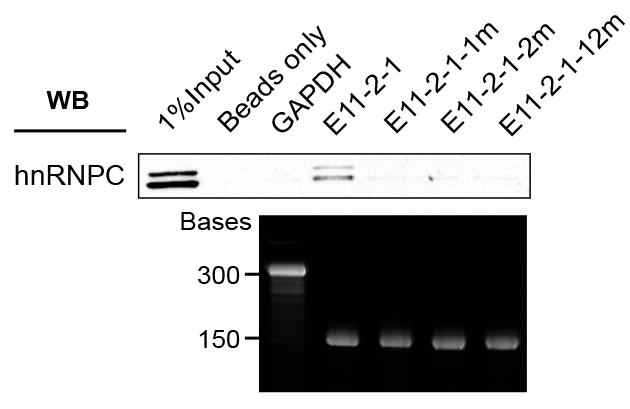


**Figure S9: Interaction between hnRNPC and the E11-2-1 sub-fragment with mutated U-tract motifs was measured by *in vitro* RNA/protein interaction assay.** Top: The binding affinity of hnRNPC to this region was detected by *in vitro RNA* pull-down assay. E11-2-1-1m represents the E11-2-1 fragment with the first U-tract mutated. E11-2-1-2m represents the E11-2-1 fragment with the second U-tract mutated. E11-2-1-12m represents the E11-2-1 fragment with all U-tracts mutated. E11-2-1 is a positive binding control. Bottom: 20% Input of RNA fragments were loaded in the 10% TBE-Urea gel (Thermo Scientific, USA) and visualized with SYBR Gold (Thermo Scientific, USA).


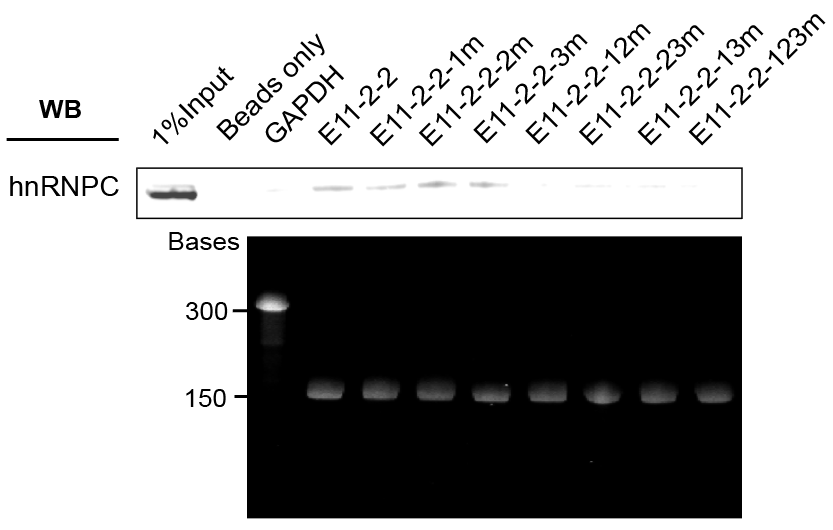


**Figure S10: Interaction between hnRNPC and the E11-2-2 sub-fragment with mutated U-tract motifs was measured by *in vitro* RNA/protein interaction assay.** Top: The binding affinity of hnRNPC to this region was detected by *in vitro RNA* pull-down assay. E11-2-2-1m represents the E11-2-2 fragment with the first U-tract mutated. E11-2-2-2m represents the E11-2-2 fragment with the second U-tract mutated. E11-2-2-3m represents the E11-2-2 fragment with the third U-tract mutated. E11-2-2-12m represents the E11-2-2 fragment with the first two U-tracts mutated. E11-2-2-23m represents the E11-2-2 fragment with the last two U-tracts mutated. E11-2-2-13m represents the E11-2-2 fragment with both the first and third U-tracts mutated. E11-2-2-123m represents the E11-2-2 fragment with all U-tracts mutated. E11-2-2 is a positive binding control. Bottom: 20% Input of RNA fragments were loaded in the 10% TBE-Urea gel (Thermo Scientific, USA) and visualized with SYBR Gold (Thermo Scientific, USA).


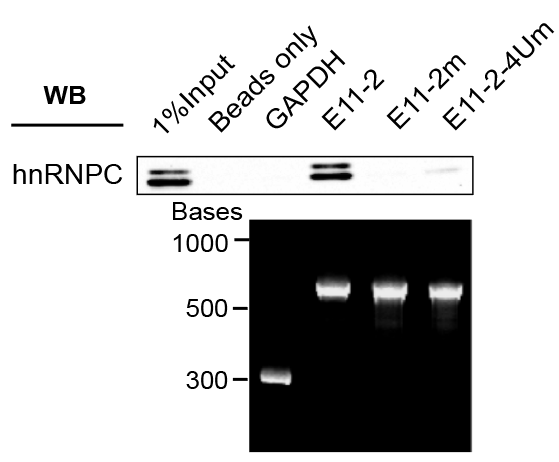


**Figure S11: Binding affinity of hnRNPC with E11-2 was mostly abolished after mutation of the first four U-tracts shown in E11-2.** Top: The binding affinity of hnRNPC to this region was detected by *in vitro RNA* pull-down assay. E11-2m represents the E11-2 fragment with all U-tracts mutated. E11-2-4Um represents the E11-2 fragment with the first four U-tracts mutated. E11-2 is a positive binding control. Bottom: 20% Input of RNA fragments were loaded in the 6% TBE-Urea gel (Thermo Scientific, USA) and visualized with SYBR Gold (Thermo Scientific, USA).


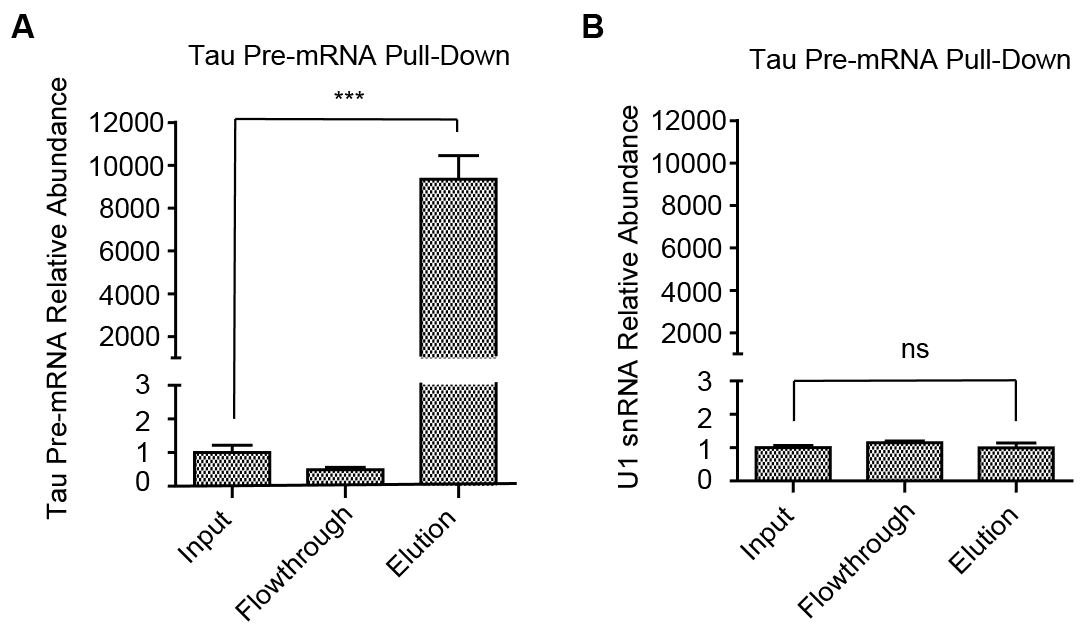


**Figure S12: Enrichment of endogenous Tau pre-mRNA in SH-SY5Y cells.** (A) Endogenous Tau pre-mRNA abundance through RAP process was measured by qRT-PCR using the primers Fp and Rp as shown in Figure 1B. (B) The U1 snRNA abundance was measured by qRT-PCR with the same samples as (A). Data shown represent the means ± SEM (n = 3, ***P < 0.001; ns: not significant, two-tailed Student's t test)
